# Effects of cancer severity on the relationship between emotional intelligence, perceived social support, and psychological distress in Italian women

**DOI:** 10.1007/s00520-024-08346-0

**Published:** 2024-02-03

**Authors:** Francesco Bruno, Chloe Lau, Carlotta Tagliaferro, Georgia Marunic, Lena C. Quilty, Marco Tullio Liuzza, Francesca Chiesi

**Affiliations:** 1 Regional Neurogenetic Centre (CRN), Department of Primary Care, ASP Catanzaro, Lamezia Terme, Catanzaro, Italy; 2 Association for Neurogenetic Research (ARN), Lamezia Terme, Catanzaro, Italy; 3 Academy of Cognitive Behavioral Sciences of Calabria (ASCoC), Lamezia Terme, Catanzaro, Italy; 4 Centre for Addiction and Mental Health, Toronto, Canada; 5 Department of Neuroscience, Psychology, Drug, and Child’s Health (NEUROFARBA), Section of Psychology, University of Florence, Florence, Italy; 6 Department of Psychiatry, University of Toronto, Toronto, Canada; 7 Department of Medical and Surgical Sciences, “Magna Graecia” University of Catanzaro, Catanzaro, Italy

**Keywords:** Emotional intelligence, Social support, Anxiety, Stress, Depression, Psychological distress, Cancer, Cancer stage, Women

## Abstract

**Purpose:**

This study aims to understand the association between emotional intelligence, perceived social support, and psychological distress (i.e., anxiety, depression, stress) in women with cancer at different stages. Specifically, the aims of this study were to investigate: i) the links between emotional intelligence and psychological distress (i.e., symptoms of anxiety, stress and depression); ii) the mediating role of perceived social support provided by family members, friends, and significant others in the relationship between emotional intelligence and psychological distress; iii) the impact of cancer type and cancer stage (I-II *vs* III-IV) in moderating these relationships, among Italian women.

**Methods:**

The research sample consisted of 206 Italian women (*mean age* = 49.30 ± 10.98 years; 55% breast cancer patients) who were administered a questionnaire to assess emotional intelligence, perceived social support, and psychological distress. Structural equation model (SEM) analysis was carried out to confirm the hypothetical-theoretical model.

**Results:**

Emotional intelligence had a positive association with perceived social support, which in turn prevented psychological distress only in women with early-stages cancers. The type of cancer has no effect on these relationships.

**Conclusions:**

The findings of this study indicate a pressing need to screen and recognize women with lower emotional intelligence and perceived social support, as they may be more prone to experiencing psychological distress. For such individuals, our results recommend the implementation of psychological interventions aimed at enhancing emotional intelligence and fortifying their social support networks, with consideration for the stage of cancer they are facing.

## Introduction

Cancer ranks among the prevalent chronic illnesses in Italy, with approximately 391,000 new cases reported in 2022 [1]. The most frequently diagnosed cancers among Italian women were breast cancer (30%), followed by colorectal (12%), lung (7.9%), endometrial (5.5%) and thyroid (4.7%) [1, 2]. According to the TNM classification, there are four main different stages of cancer: Stage I (localized cancer); Stage II (early locally advanced cancer); Stage III (late locally advanced cancer); Stage IV (metastatic cancer) [3, 4]. Staging is crucial in the diagnosis of cancer as it determines the phase of the disease, playing a vital role in choosing the most suitable treatment and influencing the prognosis [5–7]. Psychological distress, encompassing symptoms like depression, anxiety, and stress, is a well-documented issue among cancer patients, impacting survival, as reviewed elsewhere [8, 9] and in the Italian context [10, 11]. Literature shows that female sex and advanced-stages cancers are among the major risk factors for high psychological distress [12]. The widespread of COVID-19 pandemic has had an unprecedented impact on both the general population [13–16] and cancer patients [17, 18]. In addition, the interruption of medical screening due to the COVID-19 pandemic caused a significant increase in the diagnosis of advanced-stages cancers and a consequent reduction in the prevalence of early forms in Italian women [19]. This information emphasizes the significance of identifying factors that may impact psychological distress in women dealing with cancer. The goal is to implement targeted and efficient psychological interventions that can mitigate or prevent such distress.

Several studies indicate that emotional intelligence and perceived social support are two of the main protective factors against psychological distress in the general population [20, 21]. Based on the model proposed by Mayer and Salovey [22], emotional intelligence can be defined as ‘‘the ability to perceive accurately, appraise, and express emotion; the ability to access and/or generate feelings when they facilitate thought; the ability to understand emotion and emotional knowledge; and the ability to regulate emotions to promote emotional and intellectual growth" (Mayer and Salovey [22], p. 10). Thus, Salovey and Mayer [22, 23] conceptualized emotional intelligence as composed of four distinct dimensions: i) self-emotional appraisal (SEA), which consists in the appraisal and expression of own personal emotions; ii) others’ emotional appraisal (OEA), which concerns the appraisal and recognition of other people's emotions; iii) regulation of emotion (ROE), namely the ability to regulate one's emotions; iv) use of emotion (UOE), that is the manner in which emotions are used and influence thinking or cognition to facilitate problem-solving. Preliminary data indicated that higher emotional intelligence was associated both with lower psychological distress [24] and greater perceived social support [25] in cancer patients. Smith et al. [25] also reported that emotional intelligence was negatively associated with worry across the early stage of the diagnostic cancer pathway, suggesting that also the severity of the disease (i.e., the stage of the cancer) could influence the link between emotional intelligence and psychological distress. According to the stress-buffering hypothesis, the presence of perceived social support may nullify or diminish the adverse connection between quality of life and perceived stress associated with a chronic condition [26, 27]. In addition, higher perceived social support may reduce the speed of the cancer progression [28], prevent the development of infection during chemotherapy [29], and increase survival chances [30] among women. Moreover, Di Giacomo et al. [31] also found a link between increased survival rate after breast cancer diagnosis and the management of emotional weakness in Italian women.

To our knowledge, there has been no exploration of the connection between emotional intelligence, psychological distress, and perceived social support in women with cancer. On the basis of these premises, the aims of this study were to investigate: i) the relationship between emotional intelligence and psychological distress (i.e., symptoms of anxiety, stress and depression); ii) the mediating role of perceived social support provided by family members, friends, and significant others in the relationship between trait emotional intelligence and psychological distress; iii) the impact of cancer type (breast cancer *vs* other types) and cancer severity (stage I-II *vs* III-IV) in influencing these relationships among Italian women affected by cancer.

## Patients and methods

Contact information for breast cancer survivors who were eligible to participate was obtained by psycho-oncologists operating in the voluntary association “Ali Rosa”, in Italy. A cross-sectional web-based survey design was adopted to cover the entire national territory, using the free software Google Forms©. The online survey was distributed between October 25th and December 28th of 2022, after the state of emergency due to the COVID-19 Pandemic. An information letter about the purpose of the study was mailed to all patients together with a link including questionnaires on demographic-medical variables and study questionnaires. Patients were informed that participation in the study was voluntary, the survey was anonymous and confidential, and they could withdraw from the survey at any time. Additionally, an online consent form was completed by all patients. Approval for this study was obtained from the local Ethical Committee in Catanzaro (Italy). The survey included the following questionnaires:

### Wong Law emotional intelligence scale

The Italian version of the Wong Law Emotional Intelligence Scale (WLEIS) [32, 33] was used to assess emotional intelligence. All the 16-item had 5-point Likert-type response format ranging from 1 (*Strongly disagree*) to 7 (*Strongly agree*). The scale consists of four subscales: Self Emotional Appraisal (SEA), Others’ Emotional Appraisal (OEA), Use of Emotion (UOE), and Regulation of Emotion (ROE). The total WLEIS score ranges from 16 to 80, with higher scores indicating higher perceived emotional intelligence levels. In the current study, the Cronbach's alpha indicated a good internal consistency with α = 0.89 for SEA, α = 0.85 for OEA, α = 0.89 for UOE, α = 0.90 for ROE and α = 0.93 for the total score.

### Multidimensional scale of perceived social support

Perceived social support was evaluated using the Multidimensional Scale of Perceived Social Support (MSPP) [34, 35]. The scale is composed of 12 items with response options on a 7-point Likert-type scale, ranging from 1 (absolutely false) to 7 (absolutely true). The instrument measures perceived social support from family, friends, and significant others. In our sample, Cronbach’s alpha reliabilities indicated excellent internal consistency, with α = 0.95 for family, α = 0.97 for friends, and α = 0.93 for significant others.

### Depression Anxiety Stress Scales-21

Symptoms of depression, anxiety, and stress were evaluated by administering the Depression Anxiety Stress Scales-21 (DASS-21) Italian version [36, 37]. This scale is a self-report questionnaire with 21-items measuring symptoms of depression, stress, and anxiety (seven items for each subscale) based on a four-point rating scale ranging from 0 (*did not apply to me at all*) to 3 (*applied to me much, or most of the time*). A high score on each subscale indicates elevated symptoms of depression, anxiety, or stress. In the current sample, Cronbach’s Alpha for Stress and Depression subscales were excellent (α = 0.91 and α = 0.91, respectively), and good for the Anxiety subscale (α = 0.86).

## Analysis strategy

**Fig. 1:**
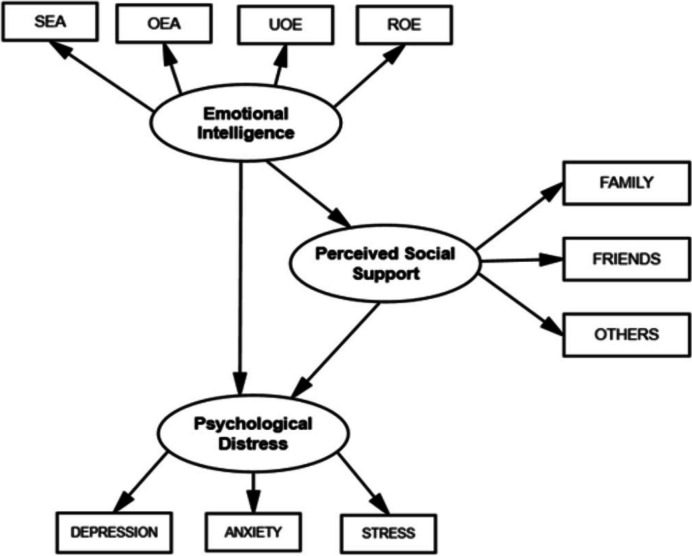
Model including emotional intelligence predicting perceived social support and psychological distress in female cancer patients. *Note*: SEA = self-emotional appraisal; OEA = others’ emotional appraisal; UOE = use of emotion; ROE = regulation of emotion

All analyses were conducted on SPSS and its extension Amos (version 27.0). Descriptive statistics and Pearson’s correlations were computed for the measured variables. Starting from the literature and the observed correlations, we tested a single-group structural equation model (SEM) that included 3 latent variables and 10 manifest variables (Fig. 1). The exogenous latent variable was emotional intelligence, perceived social support was the endogenous latent variable that mediated the relationship between emotional intelligence and psychological distress, and psychological distress was the outcome latent variable. We used the Maximum Likelihood Estimation (MLE) method to estimate the regression coefficients and the relative confidence intervals were computed with bootstrap sampling (number of bootstrap samples = 500). When bootstrap confidence intervals for the direct, indirect, and total standardized effects did not include a zero, the effects tested are supported. Goodness-of-fit was evaluated using χ2/df ratio, Comparative Fit Index (CFI), Tucker-Lewis Index (TLI), Root Mean Square Error of Approximation (RMSEA), and the Standardized Root Mean Squared Residual (SRMR). The relative chi-square should be less than 5 [38] and Byrne [39] recommended that a RMSEA and SRMR approximately above 0.08 and 0.06, and CFI and TLI above 0.90 and 0.95 would suggest moderate and excellent model fit, respectively. Modification indices (MI) were used to better specify the model. The MI is the χ^2^ value, with 1 degree of freedom, by which model fit would improve if a particular path was added. Given the size of the current sample, we used as a cutoff 10.83 that indicate the parameter would have a *p* value < 0.001, and we selected those links that ‘make sense’, i.e., that can aid to improving the model, in combination with domain or theoretical knowledge.

Multi-group SEM analysis was used to evaluate whether the model was consistent across type of cancer (contrasting breast cancer *vs* other cancers) and the severity of the disease (contrasting Stage I and II *vs* Stage III and IV). Specifically, patients with a diagnosis different from breast cancer were grouped (*n* = 116, 45%) and compared to breast cancer patients (*N* = 144, 55%). Unknown stage patients were excluded (*n* = 5) and the variable was dichotomized to obtain the Stage I and II group (*n* = 184, 68%) and the Stage III and IV group (*n* = 81, 32%). Invariance is demonstrated when the latent variables are associated with the same set of observed variables in each group (measurement invariance), and when the relationships between the latent variables are not significantly different across groups (structural invariance). To assess measurement and structural invariance, a hierarchically nested series of SEM were tested. An unconstrained model was used as a baseline (Baseline model) and compared to five more restrictive models: Model 1 (measurement parameters were constrained to be equal across groups), Model 2 (measurement and structural parameters were constrained to be equal across groups), Model 3 (structural covariances were constrained to be equal across groups), Model 4 (structural residuals were constrained to be equal across groups), and Model 5 (measurement residuals were constrained to be equal). For the sake of completeness, we tested for error/residual invariance across groups (Model 4 and Model 5), but this level of invariance may result in redundancy since the residual variance is expected to be random [36]. Models were compared using the chi-square difference statistic (Δχ2) and the comparative fit index difference (ΔCFI) and Root Mean Square Error of Approximation (ΔRMSEA) with values of ≤ 0.01 and ≤ 0.015, respectively, indicating no significant differences in nested models [40, 41]. When invariance was not achieved, we released some links to highlight the source of non-invariance between groups. Then, we repeated the analysis to test if invariance was achieved after these changes.

## Results

### Descriptives

The sample consisted of 260 women living in different Italian regions (40% Northern Italy, 25% Central Italy, 35% Southern Italy and Islands). Their age ranged from 19 to 85 years (*M* = 49.30; *SD* = 10.98) years, 49% held a high school diploma, 72% was in a relationship, and 57% was employed. Most of them were breast cancer patients (55%), who had received a diagnosis 1 to 3 years before, and about 65% were at the Stage I and Stage II of the disease. See Table 1 for the detailed socio-demographic and clinical data.

**Table 1:** Sample socio-demographic and clinical characteristics

	*N*	%
Educational status		
Primary school	4	1.5
Secondary school	34	13.1
High school	127	48.8
University	75	28.8
Master	20	7.7
Employment status		
Employed	149	57.3
Unemployed	111	42.7
Marital status		
Single	73	28.1
In a relationship	187	71.9
Diagnosis		
Breast	144	55.4
Lung	3	1.2
Colon	10	3.8
Gynecological	48	18.5
Pancreas	2	0.8
Melanoma	25	9.6
Lymphoma	9	3.5
Leukaemia	2	0.8
Kidney	7	2.7
Brain	2	0.8
Thyroid	4	1.5
Other	4	1.5
Time from diagnosis		
1 to 6 months	28	10,8
6 to 12 months	28	10,8
1 to 3 years	86	33,1
3 to 5 years	45	17,3
More than 5 years	73	28,1
Stage of the disease		
I	83	31.9
II	91	35.0
III	52	20.0
IV	29	11.2
Unknown	5	1.9
Therapy*		
Chemotherapy	167	64.2
Radiation therapy	114	43.8
Surgical	221	85.0
Other (e.g., Hormonal, Target)	129	49.6

*Some patients received more than one therapy and have more than one comorbidity. Thus. reported frequencies are the number of affirmative answers and the relative percentage on the total sample

Although small, correlations among the observed variables supported the hypothesized pattern of relationships (Table 2).

**Table 2:** Means, standard deviations, and Pearson’s correlations between the variables in the study

	*M*	*SD*	(1)	(2)	(3)	(4)	(5)	(6)	(7)	(8)	(9)
(1) Self-Emotional Appraisal	*22.45*	*4.63*									
(2) Others’ Emotional Appraisal	*22.22*	*4.14*	0.58^***^								
(3) Use of Emotion	*21.02*	*5.24*	0.54^***^	0.53^***^							
(4) Regulation of Emotion	*19.31*	*5.38*	0.53^***^	0.42^***^	0.61^***^						
(5) Perceived Social Support-Family	*5.38*	*1.68*	0.17^**^	0.24^***^	0.15^**^	0.15^*^					
(6) Perceived Social Support-Friends	*5.27*	*1.72*	0.23^***^	0.24^***^	0.22^***^	0.16^**^	0.44^***^				
(7) Perceived Social Support-Significant Others	*5.65*	*1.62*	0.19^***^	0.24^***^	0.17^**^	0.15^*^	0.65^***^	0.54^***^			
(8) Depression	*8.11*	*8.27*	-0.26^***^	-0.12^*^	-0.39^***^	-0.33^***^	-0.26^***^	-0.26^***^	-0.22^***^		
(9) Anxiety	*6.60*	*7.33*	-0.21^***^	-0.09	-0.30^***^	-0.34^***^	-0.14^*^	-0.18^**^	-0.16^**^	0.75^***^	
(10) Stress	*11.44*	*8.79*	0.25^***^	-0.08	-0.27^***^	-0.39^***^	-0.19^**^	-0.17^**^	-0.10	0.80^***^	0.72^***^

*N* = 260; *p < 0.05, **p < 0.01, ***p < 0.001.

### Single-group SEM analysis

The above-described model (Fig. 1) had an adequate fit to the data with the exception of the RMSEA value (*χ*2(32) = 96.94, *p* < 0.001, *χ*2/*df* = 3.00, CFI = 0.95, TLI = 0.92, RMSEA = 0.09), SRMR = 0.06. Modification indices suggested to include a covariance path between SEA and OEA (MI = 11.02, *p* < 0.001). Since SEA and OEA are two conceptually similar sub-scales of the WLIES scale, we added this links and the modified model showed a good fit to the data: χ2(31) = 81.70, *p* < 0.001, χ2/df = 2.64, CFI = 0.96, TLI = 0.94, RMSEA = 0.08, and SRMR = 0.06. Standardized measurement parameters were statistically significant and loaded onto its hypothesized latent variable (values ranged from 0.60 to 0.93). The structural model showed a positive association between emotional intelligence and perceived social support (β = 0.30 [90%CI: 0.15;0.45], *p* < 0.01), a negative relationship between emotional intelligence and psychological distress (β = -0.40 [90%CI: -0.51;-0.26]), *p* < 0.01), and a negative relationship between perceived social support and psychological distress (β = -0.16 [90%CI: -0.30;-0.03]). *p* < 0.05). emotional intelligence had also an indirect effect on distress (β = -0.05 [90%CI: -0.12;-0.01],* p* < 0.05). As such, the total effect of emotional intelligence on distress was β = -0.45 ([90%CI: -0.56;-0.32],* p* < 0.01). Hence, as expected, emotional intelligence had a positive effect on perceived social support, which in turn prevented from psychological distress. Moreover, emotional intelligence played a direct role in contrasting symptoms of anxiety, depression, and stress.

### Multi-group SEM analyses

Results are presented in Table 3. When comparing the model across groups defined on the type of cancer (breast cancer *vs* other cancers), goodness of fit indices supported evidence for measurement and structural invariance except for measurement residual (Δχ2 = 31.34, Δdf = 11, p < 0.01; ΔCFI = 0.016; ΔRMSEA = 0.003). As explained above, invariance at this level was not expected because it is overly strict.

**Table 3:** Invariance fit statistics across groups defined on the type of cancer diagnosis (breast *vs* other)

Model	χ^2^ (*df*)	*CFI*	*RMSEA [90% CI]*	Model comparison	Δχ2	Δ*df*	*p*	*ΔCFI*	*ΔRMSEA*
*Baseline*	147.12 (62)	0.931	0.073	*-*	-	-	-	-	-
*Model 1*	149.03 (69)	0.935	0.067	*Model 1—Baseline*	1.91	7	0.97	0.004	0.006
*Model 2*	149.45 (72)	0.938	0.065	*Model 2—Model 1*	0.42	3	0.94	0.003	0.002
*Model 3*	152.06 (73)	0.936	0.065	*Model 3—Model 2*	2.61	1	0.10	0.002	0.000
*Model 4*	155.28 (75)	0.935	0.064	*Model 4—Model 3*	3.22	2	0.20	0.001	0.001
*Model 5*	186.63 (86)	0.919	0.067	*Model 5—Model 4*	31.34	11	0.001	0.016	0.003

Breast cancer group: *N* = 144, Other cancer group: *N* = 116. χ^2^ = chi-square; *CFI* comparative fit index, *RMSEA* root mean square error of approximation, Δχ^2^ difference in chi-squares between nested models, Δ*df* difference in degrees of freedom between nested models, *p* probability value of Δχ^2^ test, ΔCFI difference between CFIs of nested models. ΔRMSEA difference between RMSEAs of nested models. Model 1 = equality of measurement weights; Model 2 = Model 1 + equality of structural weights; Model 3 = Model 2 + equality of structural covariances; Model 4 = Model 3 + equality of structural residuals; Model 5 = Model 4 + equality of measurement residuals.

When comparing the model across groups defined on the severity of the disease (Stage I/II *vs* Stage III/IV), evidence for measurement invariance was observed (Δχ2 = 9.96, Δ*df* = 7, *p* = 0.19; ΔCFI = 0.002; ΔRMSEA = 0.002). When also structural parameters were constrained to be equal across groups (Model 2), a significant change was detected when comparing Model 1 and Model 2 (Δχ2 = 15.33, Δ*df* = 3, *p* < 0.01; ΔCFI = 0.011; ΔRMSEA = 0.005). This result suggested that the estimated structural weights were not the same in the two groups. Looking at these parameters in Model 1, we can see the different values obtained for the relationships between emotional intelligence and perceived social support (0.48 *vs* -0.01 in Stage I/II and Stage III/IV, respectively), and perceived social support and psychological distress (-0.20 *vs* -0.03 in Stage I/II and Stage III/IV, respectively). Thus, these two links were freed (Model 2a) and compared to Model 1 obtaining invariance across groups (Δχ2 = 1.97, Δ*df* = 1, *p* = 0.16; ΔCFI = 0.001; ΔRMSEA = 0.000). Then, factor variances and covariances were constrained to be equal across group (Model 3) and compared to Model 2a. Invariance was observed (Δχ2 = 1.68, Δ*df* = 1, *p* = 0.20; ΔCFI = 0.000; ΔRMSEA = 0.000) as well as when Model 3 was contrasted to Model 4 (Δχ2 = 5.90, Δ*df* = 2, *p* = 0.052; ΔCFI = 0.004; ΔRMSEA = 0.001), but not for the comparison between Model 4 and Model 5 (Δχ2 = 28.30, Δ*df* = 13, *p* < 0.008; ΔCFI = 0.012; ΔRMSEA = 0.001). Results are presented in and Table 4.

**Table 4:** Invariance fit statistics across groups defined on the stage of the disease (Stage I/II *vs* Stage III/IV)

Model	χ^2^ (*df*)	*CFI*	*RMSEA [90% CI]*	Model comparison	Δχ2	Δ*df*	*p*	*ΔCFI*	*ΔRMSEA*
*Baseline*	122.59 (62)	0.950	0.062	*-*	-	-	-	-	-
*Model 1*	132.54 (69)	0.948	0.060	*Model 1—Baseline*	9.96	7	0.191	0.002	0.002
*Model 2*	147.87 (72)	0.937	0.065	*Model 2—Model 1*	15.33	3	0.002	0.011	0.005
*Model 2a*	134.51 (70)	0.947	0.060	*Model 2a—Model 1*	1.97	1	0.161	0.001	0.000
*Model 3*	136.19 (71)	0.947	0.060	*Model 3—Model 2a*	1.68	1	0.195	0.000	0.000
*Model 4*	142.09 (73)	0.943	0.061	*Model 4—Model 3*	5.90	2	0.052	0.004	0.001
*Model 5*	170.39 (86)	0.931	0.062	*Model 5—Model 4*	28.30	13	0.008	0.012	0.001

Stage I/II: *N* = 174, Stage III/IV: *N* = 81. χ2 chi-square, *CFI* comparative fit index, *RMSEA* root mean square error of approximation, Δχ2 difference in chi-squares between nested models, Δ*df* difference in degrees of freedom between nested models, *p* probability value of Δχ2 test, ΔCFI difference between CFIs of nested models. ΔRMSEA difference between RMSEAs of nested models. Model 1 = equality of measurement weights; Model 2 = Model 1 + equality of structural weights; *Model 2a* = *Model 1* + equality of structural weights only between emotional intelligence and distress. Model 3 = Model 2a + equality of structural covariances; Model 4 = Model 3 + equality of structural residuals; Model 5 = Model 4 + equality of measurement residuals

## Discussion

This study aims to understand the association between emotional intelligence, perceived social support and psychological distress (i.e., anxiety, depression, stress) in women affected by cancer.

As we expected, correlational analyses showed that all dimensions of emotional intelligence (i.e., self-emotional appraisal, others’ emotional appraisal, use of emotion, regulation of emotion) were negatively related to psychological distress (i.e., symptoms of anxiety, stress and depression), and positively related to perceived social support by family members, friends and significant others. These results are consistent with the previous studies that reported relationships between emotional intelligence and mental distress [42–44] and emotional intelligence and perceived social support [45–48] among non-clinical sample. Aligned with the study of Kong et al. [44] on Chinese young adults, we also found that emotional intelligence predicted psychological distress through the mediating effect of perceived social support in Italian females affected by cancer. In other words, women with higher levels of emotional intelligence had a propensity to perceive greater social support from others, which thus contributed to a decrease in their psychological distress. Although these relationships were not influenced by the type of cancer (i.e., breast cancer *vs* other cancers), cancer stage (I-II *vs* III-IV) affects the association between emotional intelligence and perceived social support, and between perceived social support and psychological distress. Indeed, perceived social support was not related to emotional intelligence in women with stage III and IV cancer, and it was unable to protect against the development of symptoms of anxiety, stress and depression. This result is partially consistent with the finding of Ringdal et al. [49] who reported that terminal cancer patients with high social support showed better emotional functioning and less serious stress reactions than patients with a low degree of social support only two months after baseline assessment. A possible explanation is that as the patients are getting weaker and closer to the terminal phase of their illness, social support becomes unable to mitigate the psychological distress that derives from the awareness of being in the terminal phase of the disease. Indeed, in this phase family, friends, and relevant others might start to experience the complexity of the grieving process that includes denial, anger, depression. As such, they lose their supportive and care role. Further studies are needed to confirm this hypothesis.

## The theoretical and practical contribution of the study

Given the absence of studies examining the cause-and-effect relationship among emotional intelligence, perceived social support, and psychological distress in women with cancer, our findings contribute to the theoretical understanding of this research area. Specifically, our study reveals that emotional intelligence positively influenced perceived social support, subsequently mitigating psychological distress, but only in women with early-stage cancers. The implications underscore the importance of incorporating emotional intelligence screening into a comprehensive psychological evaluation for cancer patients, enabling the identification of women with lower emotional intelligence and a potential heightened vulnerability to psychological distress. For these individuals, our results advocate for the implementation of psychological interventions focused on enhancing emotional intelligence to effectively alleviate psychological distress. For instance, preliminary data suggests the effectiveness of emotional intelligence training in reducing stigma among cancer patients [50]. Thus, our findings may inspire future research exploring the efficacy of emotional intelligence skills training in addressing symptoms of anxiety, stress, and depression in women with early-stage cancers.

## Limitations of the study

This study has limitations that offer insights for designing future research. While structural equation modeling (SEM) was utilized to explore theoretical models, data collected were cross-sectional, and future studies would benefit from adopting a longitudinal design. Additionally, self-reported measures were exclusively administered to patients. Future studies may assess the benefits of emotional intelligence in alleviating psychological distress of family, friends, and other relevant individuals. Lastly, future research might include various methods (such as clinician ratings and proxy reports) to provide a larger picture of the psychosocial challenges that patients with cancer have to face.

## Conclusion

In sum, our results suggest that emotional intelligence had a positive effect on perceived social support, which in turn prevented psychological distress in women with early-stages cancers. The type of cancer has no effect on these relationships. Further studies are needed to better characterize the relationship between emotional intelligence, perceived social support and psychological distress in women with advanced-stages cancers.

## Data Availability

All data and materials are available from the corresponding authors upon request.
